# Clinical profile and conversion rate to full psychosis in a prospective cohort study of youth affected by autism spectrum disorder and attenuated psychosis syndrome: A preliminary report

**DOI:** 10.3389/fpsyt.2022.950888

**Published:** 2022-09-23

**Authors:** Assia Riccioni, Martina Siracusano, Michelangelo Vasta, Michele Ribolsi, Federico Fiori Nastro, Leonardo Emberti Gialloreti, Giorgio Di Lorenzo, Luigi Mazzone

**Affiliations:** ^1^Child Neurology and Psychiatry Unit, Tor Vergata University Hospital, Fondazione PTV – Policlinico Tor Vergata, Rome, Italy; ^2^Chair of Child Neurology and Psychiatry, Department of Systems Medicine, University of Rome Tor Vergata, Rome, Italy; ^3^Department of Biomedicine and Prevention, University of Rome Tor Vergata, Rome, Italy; ^4^Unit of Neurology, Neurophysiology, Neurobiology, and Psychiatry, Department of Medicine, University Campus Bio-Medico of Rome, Rome, Italy; ^5^Chair of Psychiatry, Department of Systems Medicine, University of Rome Tor Vergata, Rome, Italy; ^6^Psychiatry and Clinical Psychology Unit, Fondazione PTV – Policlinico Tor Vergata, Rome, Italy; ^7^IRCCS Fondazione Santa Lucia, Rome, Italy

**Keywords:** ultra high risk, prodrome, psychosis, schizophrenia, autism, comorbidity

## Abstract

Psychosis can occur at high rates in individuals with autism spectrum disorder (ASD). However, the detection of prodromal psychotic symptoms, including attenuated psychosis syndrome (APS), conditions at high risk of converting to full psychosis, has not been extensively investigated in ASD. We longitudinally evaluate a sample of young ASD individuals (age, mean ± *SD*: 13 ± 2.9) with (*n* = 13) or without (*n* = 18) concomitant APS through a standardized assessment of autistic (Autism Diagnostic Observation Schedule–Second Edition; ADOS−2) and psychotic (Structured Interview for Psychosis-Risk Syndromes, SIPS) symptoms and cognitive and adaptive skills. Individuals with other neuropsychiatric disorders were excluded. We estimated the conversion rate to full psychosis (according to SIPS criteria) over time (39.6 ± 11.5 months) and explored the role of clinical variables at baseline in the transition to full psychosis. A conversion rate to full psychosis of 30.7% was found in ASD/APS. Conversion to full psychosis was not affected by the severity of the autistic and psychotic symptoms. At baseline, young individuals with ASD/APS who later converted to full psychosis showed lower cognitive performance (*d* = 2.05) and greater impairment of adaptive social functioning profile (*d* = 1.2) than those with ASD. The results of this preliminary report revealed that nearly a third of young individuals with ASD/APS convert to full psychosis over time. Conversion to full psychosis is affected by decreased cognitive and adaptive skills. Further investigations are needed to confirm the utility of APS detection and to better characterize the psychotic developmental trajectory in ASD, with consequent important implications on prognosis and therapeutic strategies.

## Introduction

Autism Spectrum Disorder (ASD) is a neurodevelopmental condition characterized by socio-communicative skills impairment and a set of restricted and stereotyped interests and behaviors (1). The majority of individuals with ASD are diagnosed with at least one or more accompanying psychiatric disorders during their lifetime, being at increased risk for severe mental illness and subsequent greater impairment in future outcomes (2, 3). Among psychiatric comorbidities, psychosis and schizophrenia spectrum disorders (SSDs) are described at variable rates (4, 5). A recent umbrella review on the topic reported a prevalence of 4% for SSDs in ASD (3), whereas a meta-analysis study reported a pooled prevalence of 9.4 % (95 %CI = 7.52, 11.72) for psychosis in adults with autism (6). Nevertheless, it is well-known that not only individuals with ASD are at increased risk for concomitant psychotic symptoms, but that, similarly, individuals with SSDs can exhibit greater autistic symptoms if compared to typically developing peers (7).

Even if SSDs and ASD are currently conceptualized as distinct conditions, there is strong evidence for an existing phenotypical continuum between ASD and psychosis (8, 9), with a subsequent overlap of similar clinical features, particularly referred to social skills impairments (5, 10–12). Indeed, psychotic symptoms, like social withdrawal, blunted affect, and disorganized speech can be easily disguised as ASD core symptoms such as the lack of eye contact, poor emotional and social reciprocity, reduced facial mimics, and use of gestures (11). Consequently, detection of the psychotic symptoms has proven to be particularly challenging in individuals with ASD, especially at prodromal phases (12, 13).

In this context, it is important to highlight that social skills impairment has been identified as a significant predictor of conversion to full psychosis in individuals considered at “clinical high risk” (CHR) for psychosis (14–16). Specifically, the term CHR refers to a condition characterized by subthreshold psychotic symptoms at elevated risk of developing a psychotic disorder within 3 years (17–19). Among the CHR conditions, the Attenuated Psychosis Syndrome (APS) is currently considered the most common one (20) and the best clinical predictor of conversion to full psychosis in the general population (21). Specifically, APS has been recently (2013) introduced in the Diagnostic and Statistical Manual of Mental Disorders-Fifth Edition (DSM-5)- Research Appendix section III. APS is defined by the presence of delusions, hallucinations, or disorganized speech in an attenuated form that are present at least once per week for the past month and which have never been severe enough for the individual to meet diagnostic criteria for a psychotic disorder (1). Despite the DSM-5 APS diagnosis has been a widely debated topic in the recent literature (21), some authors are concordant with the belief that APS diagnosis could encourage CHR status recognition, especially in highly specific contexts (22) and in child and adolescent populations (23, 24).

Despite evidence of an increased risk of psychosis in young individuals with ASD, to date, it is unclear whether they exhibit prodromal symptoms and conversion rates compared to the general population (25, 26). Hence, research aiming to better explore the overlap of prodromal psychotic symptoms in ASD is progressively growing (9, 25, 27, 28). Foss-Feig et al. (25), in a study conducted in the context of the NAPLS2 consortium (29), compared young (age range 12–35 years) individuals with CHR (*n* = 738) to those presenting concomitant ASD (CHR+ASD *n* = 26), and reported worse social cognition skills in individuals with autism but similar conversion rates among groups (18.2% CHR/ASD vs. 16.8% CHR no ASD). However, it is important to notice that so far, there is still a paucity of empirical longitudinal studies on the topic. To the best of our knowledge, the majority of available data are derived from retrospective studies mostly conducted on small samples (12, 25, 26), thus not permitting us to draw substantial conclusions.

The main goal of the present study was to longitudinally evaluate a sample of young individuals with ASD, with or without concomitant APS through a standardized assessment of autistic and psychotic symptoms, and cognitive and adaptive skills. Moreover, we aimed to estimate the conversion rate over time and to explore if selected clinical variables at baseline could play a role in the transition to full psychosis in individuals with ASD.

Based on the available literature on the topic, we hypothesize that ASD individuals who presented concomitant APS at baseline may maintain the APS condition or convert to full psychosis at follow-up. Moreover, we hypothesize that specific baseline variables—meant as increased psychotic and autistic symptoms severity and a lower intelligence quotient index—may be related to an increased risk for conversion in the ASD/APS group.

## Materials and methods

This is a prospective cohort study, conducted in the context of a previous research project (28). A sample of individuals with ASD (age range: 10–23 years) was longitudinally observed for a period ranging from 24 to 48 months to evaluate the presence or not of a concomitant APS status at baseline (T0) and, consequently, the conversion rates to full psychosis at follow-up (T1).

The study was approved by the Ethical Committee Board of the University of Rome Tor Vergata Hospital (#126/18) and informed consent was obtained from all legal holders of custody at both stages (T0 and T1).

### Procedure

Participants were recruited from the Children Psychiatry Unit of the University Hospital Tor Vergata of Rome. To be included in the study participants were required to have: a diagnosis of ASD according to the DSM-5 (1), supported by the Autism Diagnostic Observation Schedule–Second Edition (ADOS−2) (30). Adopted exclusion criteria were: the presence of syndromic autism, Intellectual Quotient (IQ) equal or below 70, non-fluent speech, epilepsy, and other concurrent psychiatric or neurodevelopmental conditions (i.e., Obsessive-Compulsive Disorder, Attention Deficit and Hyperactivity Disorder). In this regard, a detailed personal and family clinical interview was performed in order the exclude the presence of associated psychiatric (excepted for APS), neurological or genetic/syndromic conditions.

Thus, at baseline (T0) and at follow-up (T1) a comprehensive clinical assessment of cognitive and adaptive skills, as well as of autistic and psychotic symptoms was performed as described below.

From an initial sample of *n* = 40 ASD individuals, *n* = 3 did not complete the baseline (T0) clinical assessment and dropped out of the study. The final sample of *n* = 37 ASD (8 females, 29 males; 1:4 F/M) was included in our previous study (28). Specifically, based on the baseline (T0) clinical assessment the sample was divided into two groups: ASD (*n* = 21) and ASD/APS group (*n* = 16).

The original study design stated to follow-up ASD individuals at 24 and 36 months. Unfortunately, the follow-up time period was during the first COVID-19 pandemic era. Consequently, we needed to modify the study design and the follow-up time range. Therefore, after a time range of 24–48 months (T1) participants underwent the same clinical evaluation performed at T0, to evaluate the clinical progression and the conversion rates to full psychosis among the ASD/APS group (*ASD/APS-*: non-converters; *ASD/APS*+: converters).

From the original sample of *n* = 37 ASD, *n* = 6 (5 males and 1 female) did not perform the follow-up assessment (T1) and dropped out from the study.

#### Cognitive skills assessment

Depending on the age and each individual's ability to cooperate, all individuals with ASD underwent a non-verbal or verbal cognitive evaluation to assess their IQ at both stages (T0 and T1). Specifically, the Leiter International Performance Scale-Revised (Leiter-R) (31), which is not reliant on verbal skills, was chosen for children and adolescents with more severe communications impairments and limited levels of cooperation. Otherwise, the Wechsler Intelligence Scale for Children-fourth Edition (WISC-IV) (32) or the Wechsler Adult Intelligence Scale-Revised (WAIS-R) (33), tests including verbal language in the assessment of IQ, were used. For all of these scales, raw scores were converted into composite scores, and a mean and standard deviation (*SD*) IQ value of 100 ± 15 was considered.

#### Adaptive skills assessment

The Adaptive Behavior Assessment System, Second Edition (ABAS-II) (34) was administered to all included individuals' parents. In particular, the “5–21 years” ABAS-II form was used. Parents were asked to rate the child's skills to complete an activity (from 0 = “not able to” to 3 = “able to do it and always performs it when needed”) in regards to 10 functioning areas (i.e., communication, use of the environment, preschool competences, domestic behavior, health and safety, play, self-care, self-control, social abilities, and motility). The questionnaire provides three main adaptive domains: conceptual (CAD), practical (PAD), social (SAD), and a comprehensive score, General Adaptive Composite (GAC). Each of these indexes is standardized with a mean of 100 and an *SD* of 15.

#### Autistic symptoms assessment

The ADOS-2 (30) was administrated. The ADOS-2, a semi-structured observational assessment of autistic symptoms, includes five modules based on expressive language level and age. The ADOS-2 algorithm is organized in Social Affect (SA), Restricted and Repetitive Behaviors (RRB), and the total score (TOT). Modules 1, 2, and 3 provide the Calibrated Severity Score (CSS), ranging from 1 to 10, indicating autism severity. To compare CSS scores (35), module 3 was administrated to all individuals, following the ADOS-2 manual (30), which allows clinicians and researchers to choose module 3 also for adolescents aged over 16. In this study, the ADOS-2 was performed by two certified clinical raters (AR and MS, child and adolescent psychiatrists): specifically, one rater performed the evaluation, and the other one assisted in the assessment session and the scoring procedure. Consensus between raters was obtained by discussing the ratings and scores just after the evaluation session.

Besides, the Social Responsiveness Scale questionnaire (SRS) (36) was also administered. The SRS is a 65-item questionnaire applied to parents of children aged 4–18 years, aimed to evaluate social motivation, social awareness, social cognition, social communication, restricted interests, and repetitive behavior items. Total scores can be converted into T-scores.

#### Psychotic symptoms evaluation

Based on previous researches (28, 37), the Structured Interview for Psychosis-Risk Syndromes (SIPS) (38–40) was administrated by expert clinicians to all included individuals with ASD. The SIPS is a semi-structured interview, which rates along four major symptom dimensions on the Scale of Prodromal Symptoms (SOPS): Positive (SIPS-P), Negative (SIPS-N), Disorganized (SIPS-D), General (SIPS-G) symptoms. The SIPS/SOPS scale was administrated in order to evaluate the presence of concomitant APS at baseline (T0) and the possible conversion to full psychosis at follow-up (T1). The presence of a concomitant APS condition was considered confirmed with a score of 3, 4, of 5 on the SIPS positive symptoms scale (SIPS-P) (21, 38). Based on the SIPS Presence of Psychotic Symptoms criteria the conversion to a full psychosis was assessed when, at least, a score of 6 was acquired on the SIPS-P scale at follow-up (T1) (15, 25).

As previously reported for the assessment of the autistic symptoms, the SIPS was performed by two psychiatrists with expertise in the field of psychosis (MR and FFN). Specifically, one performed the interview, and one assisted in the evaluation session and the scoring procedure. Consensus between raters was obtained by discussing the ratings and scores just after the assessment session.

For the present study, the total scores for each SIPS subscale (total SIPS-P, total SIPS-N, total SIPS-D, total SIPS-G, and SIPS total score) were analyzed.

### Statistical analyses

The Independent sample *t*-test, Mann-Whitney test, and Median test were used to compare ASD and ASD/APS groups at baseline (T0) in terms of autistic and psychotic symptoms severity, IQ value, and adaptive skills. Therefore, the same independent sample tests were used to evaluate differences between the ASD/APS+ and ASD/APS- groups in terms of baseline clinical features (autistic and psychotic symptoms, IQ, adaptive skills) and the subsequent conversion at follow-up (T1). An alpha level of 0.05 was used for all statistical analyses, performed using SPSS v.23.0 (IBM Corp., Armonk, NY, USA). Data are presented as means ± *SD* unless otherwise specified. Finally, effect sizes (Cohen's *d*) for all the included variables were calculated.

## Results

A final sample of 31 ASD individuals was included in the study. Based on clinical assessment performed at baseline (T0), the sample was divided into two groups: ASD [*n*: 18; M/F: 15/3; age: 13.4 ± 2.91 years] and ASD/APS [*n*: 13; M/F: 9/4; age: 13.6 ± 3.12 years] (Table 1).

**Table 1:** Baseline clinical characteristics in ASD vs. ASD/APS individuals.

	**ASD** **(n = 18)**	**ASD/APS** **(n = 13)**			
	**MEAN (SD)**	**MEAN (SD)**	***p***	***t***	***Cohen's d***
Age T0	12.5 (2.91)	13.6 (3.12)	0.290	1.07	0.364
Male/Female	15/ 3	9/ 4	-	-	-
IQ	105.5 (19.5)	98 (20.1)	0.307	1.04	0.378
**ABAS-II**					
ABAS_GAC	78.28 (19.3)	63.54 (13.2)	0.024	2.530	0.891
ABAS_CAD	85.22 (17.4)	71.92 (13.8)	0.030	2.280	0.846
ABAS_SAD	80.67 (18.1)	68.08 (10.9)	0.034	2.225	0.842
ABAS_PAD	77.11 (21.6)	60.54 (15.9)	0.027	2.334	0.873
**ADOS-2**					
ADOS_SA	7.67 (2.3)	9.54 (3.1)	0.686	0.164	0.685
ADOS_RRB	1.83 (1.5)	2.23 (2.0)	0.326	0.966	0.226
ADOS_CSS	5.83 (1.6)	6.85 (2.1)	0.119	2.42	0.546
**SRS**					
SRS_T	73.61 (15.7)	81.36 (15.6)	0.207	−7.753	0.495
SRS_SA	59.89 (14.3)	70.82 (13.4)	0.051	−2.044	0.776
SRS_SC	67.94 (17.1)	74.64 (18.6)	0.330	−0.991	0.375
SRS_SCo	73.28 (11.9)	78.09 (15.5)	0.356	−4.813	0.348
SRS_SM	72.67 (14.7)	73.73 (19.2)	0.868	−1.061	0.061
SRS_AM	75.11 (16.4)	81.36 (14.9)	0.313	−6.253	0.398
**SIPS**					
SIPS-P	2.67 (1.53)	6.62 (2.3)	<0.001	17.29	2.02
SIPS-N	2.39 (1.57)	5.38 (4.2)	0.291	1.11	0.943
SIPS-D	1.28 (1.48)	4.00 (2.8)	<0.001	11.49	1.21
SIPS-G	1.96 (1.05)	2.92 (2.6)	0.119	2.42	0.484
SIPS-Total	7.39 (3.61)	18.85 (9.7)	<0.001	23.88	1.56

IQ, Intelligent Quotient; ABAS-II, Adaptive Behavior Assessment System, Second Edition; ABAS_GAC, ABAS General Adaptive Domain; ABAS_CAD, ABAS Conceptual Adaptive Domain; ABAS_SAD, ABAS Social Adaptive Domain; ABAS_PAD, ABAS Practical Adaptive domain; ADOS-2, Autism Diagnostic Observation Schedule – Second Edition, ADOS-2; ADOS_SA, Social Affect; ADOS_RRB, Restricted and Repetitive Behaviors; ADOS_CSS, Calibrated Severity Score; SRS, Social Responsiveness Scale; SRS_T, SRS Total Score; SRS_SA, SRS social awareness; SRS_SC, SRS social cognition; SRS_SCo, SRS social communication; SRS_SM, SRS social motivation; SRS_AM, SRS autistic mannerism; SIPS, Structured Interview for Psychosis-Risk Syndromes; SIPS-P, total positive symptoms domain; SIPS-N, total negative symptoms domain; SIPS-D, total disorganization symptoms domain; SIPS-G, total general symptoms domain.

df = 29.

### Baseline (T0) clinical profiles characteristics

At baseline (T0), there were no statistically significant differences between ASD and ASD/APS in terms of age (*t* = 1.07, *df* = 29, *p* = 0.29, Cohen's *d* = 0.364), IQ (*t* = 1.04, *df* = 29, *p* = 0.31, *d* = 0.378,), or ADOS-2 scores (SA: *U* = 154.5, *p* = 0.69, *d* = 0.68; RRB: *U* = 126.0, *p* = 0.33, *d* = 0.23; CSS: *U* = 158.5, *p* = 0.12, *d* = 0.55). The median ADOS-CSS score was 8 in the ASD/APS group and 6 in the ASD group. Likewise, the ASD/APS group presented with higher SRS social awareness (ASD vs. ASD/APS: 59.89 ± 14.3 vs. 70.82 ± 13.4) and social cognition (ASD vs. ASD/APS: 67.94 ± 17.1 vs. 74.64 ± 18.62) scores. The differences were not statistically significant (*t* = −2.044, *df* = 27, *p* = 0.05 and *t* = −0.991, *df* = 27, *p* = 0.33, respectively), even if a quite large effect size came out in the SA domain (*d* = 0.78).

**Figure 1:**
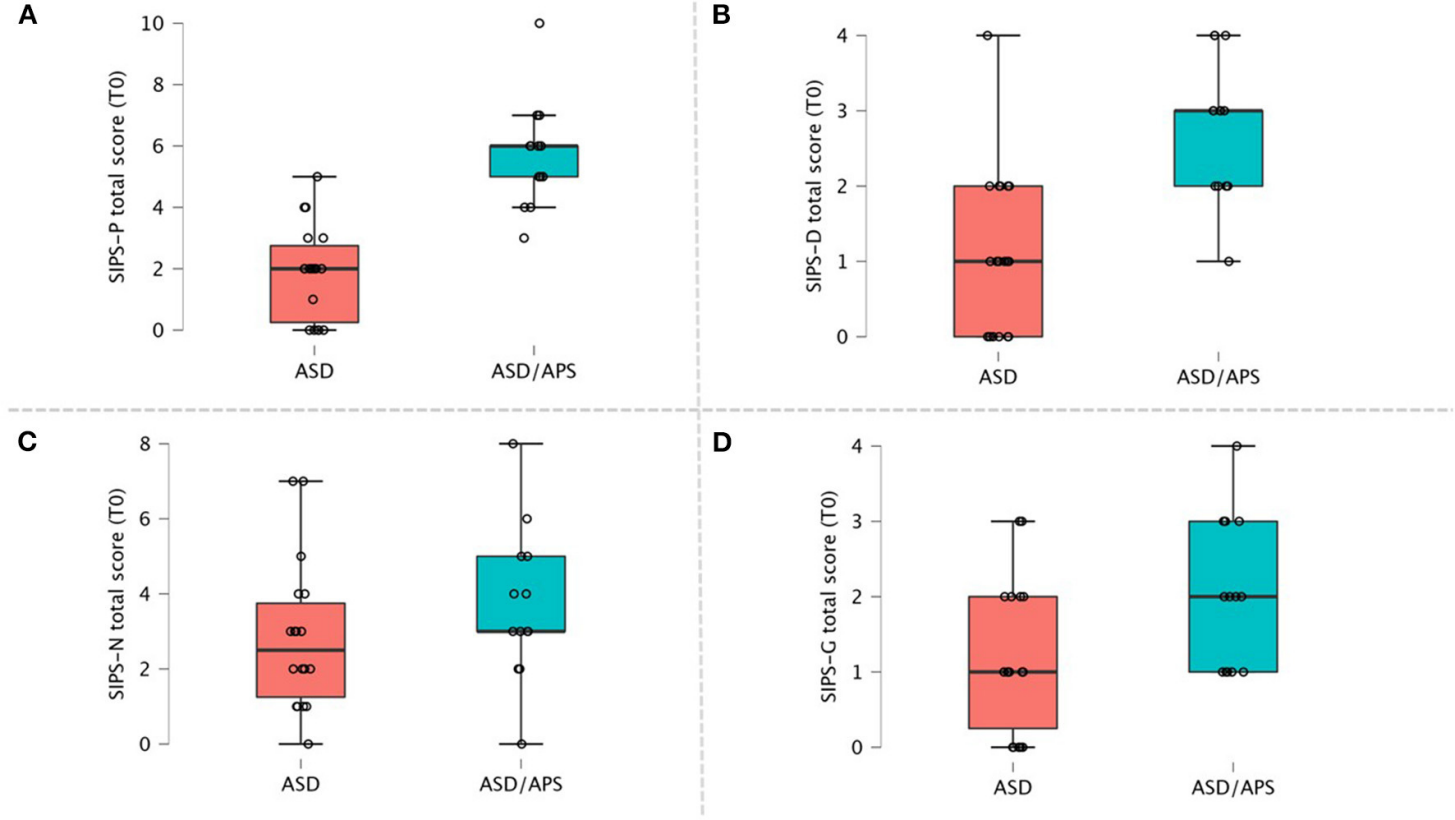
SIPS scores domain within ASD and ASD/APS individuals. **(A)** SIPS Positive symptoms total score; **(B)** SIPS Disorganized symptoms total score; **(C)** SIPS Negative symptoms total score; **(D)** SIPS General symptoms total score.

In terms of psychotic symptoms, the ASD/APS group, compared to the ASD one, exhibited higher scores in the total SIPS interview score (SIPS total: *t* = 23.88, *df* = 29, *p* < 0.001, *d* = 1.56). When considering the sub-scores, the ASD/APS group presented increased positive (SIPS-P: *t* = 17.29, *df* = 29, *p* < 0.001, *d* = 2.02) and disorganized (SIPS-D: *t* = 11.49, *df* = 29, *p* = 0.001, *d* = 1.21) symptoms levels, with no significant differences in terms of negative (SIPS-N: *t* = 1.11, *df* = 29, *p* = 0.29, *d* = 0.94) and general (SIPS-G: *t* = 2.42, *df* = 29, *p* = 0.12, *d* = 0.48) symptom severity (Figure 1).

Finally, when comparing the adaptive functioning profiles, we observed in the ASD/APS group a statistically significant more severe impairment in all the ABAS-II questionnaire domains (Table 1).

### Follow-up (T1) clinical profiles and conversion rate to psychosis

Follow-up results are summarized in Table 2. Overall, 4 out of 13 individuals with ASD/APS (M/F: 2/2) converted to psychosis (ASD/APS+) at follow-up (2 M and 2 F). No individuals with ASD without APS converted to full psychosis. Therefore, the conversion rate was 30.7% (mean T0-T1 difference: 39.6 ± 11.5 months). There were no statistically significant differences in terms of age among the individuals with ASD/APS who converted and who did not (ASD/APS+ vs. ASD/APS-:14.5 ± 5.6 vs. 13.2 ± 1.3; *t* = −0.665, *df* = 11, *p* = 0.52).

**Table 2:** ASD/APS- vs. ASD/APS+ sample characteristics.

	**ASD/APS−** **(n = 9; 69%)**	**ASD/APS+** **(n = 4; 31%)**			
	**MEAN (SD)**	**MEAN (SD)**	***p***	***t***	***Cohen's d***
Age T1	13.2 (1.3)	14.5 (5.6)	0.520	−0.665	0.319
**Baseline characteristics**					
IQ	106.5 (18.1)	78.7 (6.4)	0.014	2.926	2.047
**ABAS-II**					
ABAS_GAC	66.9 (11.1)	56.0 (16.1)	0.178	1.440	0.788
ABAS_CAD	74.5 (13.2)	66.0 (15.5)	0.326	1.028	0.590
ABAS_SAD	71.6 (10.5)	60.0 (7.7)	0.074	1.974	1.259
ABAS_PAD	62.4 (15.7)	56.2 (17.9)	0.542	0.629	0.368
**SIPS**					
SIPS-P	6.1 (1.7)	4.7 (1.5)	0.237	10.5	0.873
SIPS-N	3.2 (2.2)	4.7 (1.3)	0.099	28.5	0.830
SIPS-D	2.6 (0.7)	3.0 (1.4)	0.418	23.0	0.361
SIPS-G	2.2 (0.9)	1.5 (1.0)	0.195	10.0	0.735
SIPS Total	14.2 (2.2)	14.0 (2.5)	0.873	17.0	0.084
**ADOS-2**					
ADOS_SA	9.2 (2.2)	10.2 (5.1)	0.606	−0.531	0.254
ADOS_RRB	2.1 (2.1)	2.5 (1.9)	0.762	−0.310	0.194
ADOS_CSS	6.6 (2.1)	7.2 (2.4)	0.660	−0.452	0.266

ASD/APS-, non-converters; ASD/APS+, converters; IQ, Intelligent Quotient; ABAS-II, Adaptive Behavior Assessment System, Second Edition; ABAS_GAC, ABAS General Adaptive Domain; ABAS_CAD, ABAS Conceptual Adaptive Domain; ABAS_SAD, ABAS Social Adaptive Domain; ABAS_PAD, ABAS Practical Adaptive domain; SIPS, Structured Interview for Psychosis-Risk Syndromes; SIPS-P, total positive symptoms domain; SIPS-N, total negative symptoms domain; SIPS-D, total disorganization symptoms domain; SIPS-G, total general symptoms domain; ADOS-2, Autism Diagnostic Observation Schedule – Second Edition, ADOS-2; ADOS_SA, Social Affect; ADOS_RRB, Restricted and Repetitive Behaviors; ADOS_CSS, Calibrated Severity Score.

df = 11.

To note, a statistically significant differences came out in terms of IQ (*t* = 2.926, *df* = 11, *p* = 0.01) value between ASD/APS+ and ASD/APS-, meaning that individuals with ASD/APS who later converted to psychosis presented lower IQ scores at T0 (ASD/APS+: 78.7 ± 6.4) when compared to those who did not convert (ASD/APS-: 106.5 ± 18.1) showing a large effect size (*d* = 2.05).

When analyzing the adaptive functioning profiles at baseline in relation to the conversion rate at T1 in the ASD/APS group, there was no statistically significant difference in any ABAS-II domain between individuals who converted and those who did not (GAC: *t* = 1.440, *df* = 11, *p* = 0.18; CAD: *t* = 1.028, *df* = 11, *p* = 0.33; SAD: *t* = 1.974, *df* = 11, *p* = 0.07; PAD: *t* = 0.629, *df* = 11, *p* = 0.54). Nevertheless, in quantitative terms, at T0, the ASD/APS+ group presented with higher scores in the GAC (ASD/APS+ vs. ASD/APS-: 56 ± 16.1 vs. 66.9 ± 11.1) and SAD (ASD/APS+ vs. ASD/APS-: 60 ± 7.7 vs. 71.6 ± 10.5) domains, showing large effect sizes (GAC *d* = 0.8; SAD *d* = 1.2). Among the ASD/APS+ groups, no statistically significant differences were observed in terms of autistic (ADOS-2 SA: *t* = −0.531, *df* = 11, *p* = 0.61; ADOS-2 RRB: *t* = –0.310, *df* = 11, *p* = 0.76; ADOS-2 CSS: *t* = –0.452, *df* = 11, *p* = 0.66) and psychotic (SIPS-P: *U* = 10.5, *p* = 0.24; SIPS-N: *U* = 28.5, *p* = 0.09; SIPS-D: *U* = 23.0, *p* = 0.42; SIPS-G: *U* = 10.0, *p* = 0.19; SIPS tot: *U* = 17.0, *p* = 0.97; *df* = 11) symptoms severity, assessed at baseline (T0) and the subsequent conversion to full psychosis at follow-up (T1).

## Discussion

The primary goal of our study was to characterize the clinical profile of a sample of young individuals with ASD presenting or not concomitant APS through standardized assessment of autistic and psychotic symptoms in addition to cognitive and adaptive skills. Furthermore, we longitudinally evaluated the conversion rate to psychosis over time and if selected baseline clinical variables could have a role in the transition to full psychosis in the ASD/APS ones.

### ASD vs. ASD/APS: A comparison of clinical phenotypes

At first glance, our results did not demonstrate statistically significant differences in the clinical phenotype—meant as social skills impairment (ADOS-2, SRS), IQ, and adaptive skills deficit—between ASD individuals presenting or not concomitant APS. Specifically, concerning the social domain, our results are concordant with the general knowledge that social skills deficit is a key feature for both, ASD and psychosis (25, 41, 42). Nevertheless, when considering effect sizes and mean scores values, subtle differences emerged between groups (ASD and ASD/APS). Indeed, our results demonstrated not only a higher median ADOS-2 symptoms severity score (ASD/APS CSS:8 vs. ASD CSS:6) but also worse SRS social awareness (SA) and social cognition (SC) performances in the ASD/APS group (showing a large effect size), meaning that autistic individuals who present concomitant APS are more likely to show greater impairment in the social skills domain when compared to the ASD group. Accordingly, available data yet reported a worse baseline social performance in the ASD/APS individuals in comparison to those with APS in the general population (10, 25). These findings together may support the hypothesis of a more prominent social skills deficit in ASD/APS than would be expected in ASD and APS alone.

Furthermore, although no statistically significant differences emerged in terms of IQ value between groups (ASD vs. ASD/APS), our data showed worse adaptive skills in the ASD/APS individuals when compared to the ASD, revealing that the presence of concomitant attenuated psychotic symptoms in autistic individuals contributes not only to greater impairment in the social abilities but also in the general adaptive functioning profile.

Finally, specifically concerning the assessment of the psychotic symptoms, our data demonstrated that the main differences between ASD/APS and ASD were in the positive (SIPS-P) and disorganized (SIPS-D) symptoms domain, with no statistically significant differences in terms of negative (SIPS-N) and general (SIPS-G) symptoms. Accordingly, a growing body of evidence it's starting to support the knowledge that symptoms overlap between ASD and psychotic disorders is more prominent among negative symptoms rather than in the positive ones (13, 43).

By contrast, differentiating psychotic negative symptoms in autistic individuals still represents a challenging task, even for expert clinicians (13, 43). Indeed, our data support the evidence for reduced reliability of available tools (i.e., SIPS/SOPS) in the detection of negative symptomatology in the autistic population (25, 43), strongly supporting the need for diagnostic instruments specifically designed for individuals with ASD, with subsequent important implications in terms of differential diagnosis, clinical prognosis, and therapeutic strategies.

### ASD/APS: Follow-up assessment and conversion rate to full psychosis

Noteworthy, our results showed a conversion rate to full psychosis of 30.7% in the ASD/APS population at follow-up assessment (mean distance of 3 years from baseline). These data are discordant with a recent meta-analysis by Vaquerizo-Serrano et al. (9) that shows lower conversion rates at 2 years, ranging from 15.4 to 18.2%.

However, as suggested by Ziermans et al. (44), Vaquerizo-Serrano et al. (9) results may have been influenced by several methodological issues, which in turn do not permit an accurate comparison with our study. Specifically, the main limits of the studies included in the meta-analysis are represented by: (1) the use of overlapping study samples (considered by Vaquerizo-Serrano et al. as different samples); (2) data were mainly collected retrospectively from clinical databases of the CHR populations (25, 26), not including an assessment of ASD core symptoms through gold-standard measures (i.e., ADOS-2) with subsequent possible under/misdiagnosis of ASD in the CHR population (25); (3) the presence of different time ranges between baseline and follow-up evaluation. To note, the use within our study of a larger time difference between T0 and T1 assessment (mean 39 vs. 24 months, as reported in the previous studies) may have allowed us to increase the chance of detecting the conversion to full psychosis in the ASD/APS group. As an element of proof that these variables influence study results, other researches on the CHR population showed a conversion rate of 25–35% when considering a time frame of 3 years follow-up (20, 45).

Further longitudinal studies based on a replicable methodology and using standardized tools are needed to clarify whether ASD/APS individuals have a higher risk of conversion compared to APS without ASD and explore whether the clinical phenotype could impact this risk.

Within this framework, we evaluated if selected baseline clinical characteristics—particularly referred to the autistic and psychotic symptoms level and the IQ value—have influenced the conversion to full psychosis at follow-up in our sample. Surprisingly, no statistically significant relation between the severity of the autistic and psychotic symptoms at baseline and the subsequent conversion to psychosis came out. Our data are in contrast with the ones reported in the general population (CHR individuals), which outlined a greater social skills impairment (14, 15) and a higher psychotic symptoms level (46, 47) as significant predictors of conversion to full psychosis. Nonetheless, it is important to take into account that, to the best of our knowledge, this study is the first investigating the correlation between autistic and psychotic symptoms levels—assessed through standardized measures (ADOS-2, SIPS/SOPS)—with the subsequent psychotic conversion in ASD/APS individuals. In this regard, from a clinical point of view, our data highlighted a greater impairment in the adaptive functioning profile (ABAS-II GAC), especially in the social domain (ABAS-II SAD), in the ASD/APS individuals who later convert. Thus, based on our results, we may hypothesize that even if the severity of the autistic as well as the psychotic symptoms level at baseline seems to not effectively impact the psychotic developmental trajectory in ASD individuals, greater impairment in the adaptive functioning profile at baseline, particularly referred to the social domain, emerged in those individuals who later converted.

To note, a statistically significant difference between the baseline IQ value and the subsequent conversion in the ASD/APS group emerged. Indeed, our data highlighted that those ASD/APS individuals who presented lower IQ values at baseline were more likely to later convert to full psychosis (showing a large effect size of 2.05). While no comparable data are currently available for the ASD/APS population, the correlation between intelligence skills and psychotic risk has been described inconsistently in the general population. A recent meta-analysis in the field revealed that CHR individuals showed greater cognitive impairment in comparison to healthy controls (48); however, it is still not clear whether significant differences may exist between the converters and non-converters population (29).

Even if our results need to be interpreted with caution given the small ASD/APS+ sample size, our preliminary results could start to provide a more informative characterization of the ASD/APS individuals who later convert or do not to full psychosis, strongly underlining the need for further investigations on the topic.

### Limitations and strengths of the study

Despite some points of strength, our study presents several limitations that need to be thoroughly discussed before interpreting our data. The major limitation is the small number of enrolled subjects and, consequently, its limited statistical power. Consequently, this study should be considered a preliminary report. Nevertheless, the effect sizes for all the included variables showed, at baseline and follow-up, some moderate to large results, confirming the clinical implication of this study. However, we cannot exclude confounding factors, such as the gender effect. Furthermore, it is important to highlight that we have included individuals without intellectual and/or language impairment as well as without psychiatric comorbidity (except for APS). As a consequence, although the inclusion of “selected” individuals with ASD (without neuropsychiatric comorbidities except APS) allowed us to better explore the APS in ASD, it is also possible that it may have contributed not only to the small sample size but also to a possible recruitment bias. The lack of an APS control group without ASD is another important limitation: the exclusion of a control group did not consent to thoroughly understand differences in the psychotic developmental trajectory within ASD and the general population. Finally, the incoming of the COVID-19 pandemic era forced us to modify the original study design and the scheduled follow-up time period. Despite these limitations, we would like to underline that such a prospective study design poses some challenges to the researchers. Firstly, the enrollment of ASD participants with well-established compliance. Secondly, the clinical assessment through standardized tools particularly referred to the assessment of the psychotic symptoms in individuals with ASD, which has proved to be challenging in daily clinical practice. In this regard, to the best of our knowledge, this is the first empirical study aimed to longitudinally evaluate APS in ASD young individuals with a comprehensive clinical evaluation of cognitive and adaptive skills in addition to autistic and psychotic symptoms through standardized tools performed by a multidisciplinary team of expert clinicians in the fields of both, autism and psychosis. Moreover, we believe that the inclusion of ASD individuals with a wide age range (10–23 years), allowed us to assess the presence of psychosis risk at a very early prodromal phase (49) and during the young-adult age.

## Conclusions

To sum up, our data highlighted some interesting points. First, our preliminary findings revealed that nearly a third of young individuals with ASD/APS convert to full psychosis over time. To note, conversion to full psychosis was not affected by the severity of the autistic and psychotic symptoms, but by decreased cognitive and adaptive skills. Consequently, further investigations based on wider samples and rigorous methodological standards, including experts in the field of both, ASD and APS/psychosis, are urgently needed, in order to deeply understand the psychotic developmental trajectory in ASD individuals with subsequent crucial implications in terms of clinical prognosis and therapeutic strategies.

## Data availability statement

The data that support the finding of this study are available on request from the corresponding authors, GDL and AR.

## Ethics statement

The studies involving human participants were reviewed and approved by Policlinico Tor Vergata, Rome. Written informed consent to participate in this study was provided by the participants' legal guardian/next of kin.

## Author contributions

LM and GDL conceived and designed the present study. AR wrote the original draft. AR, MS, and MV performed the ASD assessment and the data curation. MR and FFN evaluated the psychotic symptoms. LEG performed the data analysis. LM, MS, LEG, and GDL performed the supervision and substantially revised the manuscript. All authors contributed to the article and approved the submitted version.

## Conflict of interest

The authors declare that the research was conducted in the absence of any commercial or financial relationships that could be construed as a potential conflict of interest.

## Publisher's note

All claims expressed in this article are solely those of the authors and do not necessarily represent those of their affiliated organizations, or those of the publisher, the editors and the reviewers. Any product that may be evaluated in this article, or claim that may be made by its manufacturer, is not guaranteed or endorsed by the publisher.
